# PCR detection of malaria parasites in desiccated Anopheles mosquitoes is uninhibited by storage time and temperature

**DOI:** 10.1186/1475-2875-11-193

**Published:** 2012-06-10

**Authors:** Mark A Rider, Brian D Byrd, Joseph Keating, Dawn M Wesson, Kevin A Caillouet

**Affiliations:** 1 Department of Tropical Medicine, Tulane University, 1430 Tulane Avenue, SL-17, New Orleans, LA, USA, 70112; 2 Environmental Health Sciences Program, Western Carolina University, 156 Joyner Drive, Cullowhee, NC, USA, 28723; 3 Department of Global Health Systems and Development, Tulane University, 1440 Canal Street, Suite 2200, New Orleans, LA, USA, 70112; 4 Department of Biostatistics, Virginia Commonwealth University, 830 East Main Street, 980032, Richmond, VA, USA, 23298

**Keywords:** *Plasmodium* parasite detection, DNA, Temperature, Desicant, *Anopheles*, Storage

## Abstract

****Background**:**

Reliable methods to preserve mosquito vectors for malaria studies are necessary for detecting *Plasmodium* parasites. In field settings, however, maintaining a cold chain of storage from the time of collection until laboratory processing, or accessing other reliable means of sample preservation is often logistically impractical or cost prohibitive. As the *Plasmodium* infection rate of *Anopheles* mosquitoes is a central component of the entomological inoculation rate and other indicators of transmission intensity, storage conditions that affect pathogen detection may bias malaria surveillance indicators. This study investigated the effect of storage time and temperature on the ability to detect *Plasmodium* parasites in desiccated *Anopheles* mosquitoes by real-time polymerase chain reaction (PCR).

****Methods**:**

Laboratory-infected *Anopheles stephensi* mosquitoes were chloroform-killed and stored over desiccant for 0, 1, 3, and 6 months while being held at four different temperatures: 28, 37, -20 and -80°C. The detection of *Plasmodium* DNA was evaluated by real-time PCR amplification of a 111 base pair region of block 4 of the merozoite surface protein.

****Results**:**

Varying the storage time and temperature of desiccated mosquitoes did not impact the sensitivity of parasite detection. A two-way factorial analysis of variance suggested that storage time and temperature were not associated with a loss in the ability to detect parasites. Storage of samples at 28°C resulted in a significant increase in the ability to detect parasite DNA, though no other positive associations were observed between the experimental storage treatments and PCR amplification.

****Conclusions**:**

Cold chain maintenance of desiccated mosquito samples is not necessary for real-time PCR detection of parasite DNA. Though field-collected mosquitoes may be subjected to variable conditions prior to molecular processing, the storage of samples over an inexpensive and logistically accessible desiccant will likely ensure accurate assessment of malaria parasite presence without diminishing PCR-detection of parasites in mosquitoes stored for at least six months.

## **Background**

Current estimates suggest that nearly half of the global population is at risk of malaria infection [1]. Because the malaria parasite (*Plasmodium* spp.) is vectored by anopheline mosquitoes, active vector surveillance plays a significant role in malaria prevention and control campaigns [1]. As such, sensitive methods for parasite detection and identification in mosquito vectors remain critical for effective surveillance programs.

Techniques to detect *Plasmodium* parasites in mosquitoes include, enzyme-linked immunosorbent assay (ELISA) [2], deoxyribonucleic acid (DNA) hybridization [3], direct observation by microscopy [4] and the polymerase chain reaction (PCR) [5,6]. PCR is perhaps the most commonly used technique, as it is more sensitive than others methods and less susceptible to false positive results compared to ELISA [5,7].

PCR assays require that the targeted DNA remains intact and detectable at the time of testing. However, tropical weather conditions and unreliable electricity in many malarious regions present challenges to sample preservation and parasite detection. Transport and storage of samples without the use of a robust preservation method, such as freezing on dry ice or liquid nitrogen, may result in DNA modifications or deterioration through inter-strand cross-linking, shearing, or enzymatic cleavage [8,9]. As field conditions may contribute to sample deterioration, they may also prevent the subsequent molecular detection of mosquito-borne pathogens in the laboratory. Field studies investigating malaria transmission often take place over extended periods of time [10]. This exposes early-collected samples to longer storage times than those collected later. These samples may exhibit a differential loss of DNA integrity and detection sensitivity by PCR methods. Variations in storage conditions therefore may bias temporal comparisons, and affect calculations of critical surveillance indices such as entomological inoculation rates.

Silica desiccant is widely used to preserve dipteran specimens to reduce the negative effects of humidity [11-14]. Nevertheless, it is still unclear what effect temperature and time have on PCR detection of parasite DNA in desiccated dipterans, as there have been few attempts to quantify these changes. Cooper [15] observed that mosquito DNA was less detectable by DNA probes in dried mosquitoes stored at 26°C after six months; Post and colleagues [16] observed that 35% less DNA could be extracted from *Simulium damnosum* samples held over desiccant at ambient temperature after approximately five years. Dean and colleagues [17] observed that PCR amplification of large DNA targets (> 1,000 base pairs) in naphthalene-preserved *Drosophila* museum specimens was inhibited after two years, but smaller targets were not. These observations suggest that independent of the effects of humidity, time and temperature may in some circumstances negatively affect the integrity of DNA. Though these studies indicate a loss of the ability to detect dipteran DNA under certain conditions, there is no published research describing the loss of the ability to detect malaria parasite DNA by PCR in desiccated mosquitoes.

To test the hypothesis that time and temperature deleteriously affect the quantity of PCR-detectable *Plasmodium* DNA and potentially detection sensitivity, changes in parasite DNA detection were compared in desiccated mosquitoes using real-time PCR. This diagnostic system was employed at different time and temperature intervals to detect *Plasmodium berghei* in infected, desiccated *Anopheles stephensi* mosquitoes.

## **Methods**

Live *An. stephensi* mosquitoes infected with *P. berghei* (NK65 strain), a well-established model organism for the study of human malaria [18,19], were obtained from New York University School of Medicine through a program administered by the Malaria Research and Reference Reagent Resource (MRA-887; MR4, Manassas, VA) [20]. The live mosquitoes were held overnight in a controlled environment maintained at 80% humidity and 27°C, with a 16h:8h light:dark cycle. A 10% sucrose solution was provided for nutrition. The following day (day 0), mosquitoes were chloroform killed and then placed into individual 2 mL cryogenic vials containing approximately 350 mg of Drierite® desiccant (W.A. Hammond Drierite Co, Xenia, Ohio) and a small cotton plug. To minimize potential differences in parasite detection due to parasitaemia variation, mosquitoes were randomly assigned to a time and temperature group, with ten replicates per group; two groups had 5 replicates (day 90 and day 180 groups, maintained at 37°C). Holding times were 0, 30, 90 and 180 days. Temperature conditions were -80, -20, 28, and 37°C. The control group (processed on day 0) consisted of 30 mosquitoes.

At the end of each time point, each temperature cohort was removed and dissections were performed by first removing the legs and wings. A single incision was next made between the meso- and metathoracic segments, separating the head and salivary glands from the abdomen. This is typically done with field-collected specimens to isolate mosquitoes with parasites in the midgut (*i.e.*: infected mosquitoes) from those with sporozoites in the salivary glands (*i.e.*: infectious mosquitoes). DNA was extracted from the head and proximal thorax of the individual mosquitoes using DNAzol (MRC. Inc., Cincinnati, Ohio) with the following modifications to the manufacturer’s recommendations: Individual mosquitoes were homogenized in 200 μL DNAzol by applying approximately 10 strokes to the tissue with a pestle. The homogenate was then incubated at 55 °C for 20 minutes to facilitate tissue lysis. Samples were then centrifuged for 10 minutes at 13,000 × g, and 170 μL of the resulting supernatant was retained. Three μL of PolyAcryl carrier reagent (MRC Inc., Cincinnati, Ohio) was added to the centrifuged lysate to maximize DNA recovery, and this solution was mixed and incubated for two minutes. DNA was precipitated by adding 100 μL of 100% ethanol. The solution was then mixed by inversion and stored for three minutes. The precipitate was pelleted by centrifugation for eight minutes at 5,500 × g and the DNA pellet was washed twice with 0.8 mL of 75% ethanol. Finally, the DNA was re-suspended in 100 μL of nuclease free water. The extracts were stored at -80°C until assayed by real-time PCR. All steps of the extraction were carried out at room temperature unless noted otherwise.

Real-time PCR amplification was performed with the Bio-Rad iCycler using 2X iQ™ SYBR Green Supermix (Bio-Rad, Hercules, California). PCR primers were designed to amplify a 111 base pair region of block 4 of *P. berghei* strain NK65 merozoite surface protein-1 (MSP-1) gene (Genbank Accession: AF000411) [21]. Each of the forward (5´-ACGATGATATAGATCAAAT-3´) and reverse (5´TACCTAAGCTTCTTGCGTA-3´) primers were added to a final concentration of 240 nM, along with 2 μL of extracted genomic DNA for a final reaction volume of 25 μL. Each specimen sample was assayed in triplicate. A standard dilution series was included with each PCR run and the threshold for determining the threshold cycle values (C_t_ values) was set based on the standard curve. This ensured consistent amplification between experimental cohorts and maximized the comparability of samples. The reaction efficacy was between 99 and 100% for amplification through forty cycles. As efficiency waned after forty cycles, any samples that failed to amplify before this point were deemed negative for parasite presence. The thermal cycler was programmed for forty cycles of denaturation, annealing, and extension as follows: 95°C, 59.7°C, and 72°C each for thirty seconds, respectively, followed by melt temperature analysis to ensure that the target was the sole product amplified. Single amplicons were also confirmed by agarose gel electrophoresis; no non-target amplification or primer-dimer was observed.

Primer design was performed using the web-based Primer3 utility [22]. DNA sequences were analysed with the Accelrys Gene software version 2.0 (Accelrys, San Diego, California), and the real-time PCR amplification was measured with the Bio-Rad iQ5 software version 2.0 (Bio-Rad, Hercules, California). The resulting C_t_ values were assessed statistically using a two-way factorial analysis of variance (ANOVA). This was followed by one-way ANOVA analyses of individual variables, and the Bonferroni multiple comparison tests. The adjusted coefficient of determination (*R*^2^) was used to assess the contribution of the variables tested to the variation observed in the statistical models. All statistical analyses were performed using STATA 10 statistical software (Stata Corp LP, College Station, Texas). For all statistical analyses the alpha value was set at 0.05.

## **Results**

The expected *Plasmodium* infection rate of *An. stephensi* used in this study was 90-100% [20]. This was confirmed by real-time PCR: 90.7% of mosquitoes (127 of 140) in the control group amplified *P. berghei* DNA in less than 40 cycles of PCR. Threshold cycle values ranged from 26.6 to 34.5 in the control group, corresponding to a parasitaemia of approximately 10,000 to 20,000 sporozoites per mosquito [20]. Mosquito samples that failed to realize the amplification threshold by 40 cycles were considered to be uninfected. As PCR-negative detection rates did not exceed expected negative rates, detection sensitivity was determined to be unaffected by storage time and temperature.

Of the 140 mosquitoes harvested over the course of the six-month experiment, 30 were harvested immediately (day 0) to establish the baseline ability to detect parasite DNA over time. The average C_t_ values and 95% confidence intervals at each time point were as follows: time 0, 29.86 (29.07-30.65); one month, 29.82 (28.87-30.77); three months, 29.55 (28.24-30.86); and six months, 28.37 (27.32-29.41). No association was observed between storage time and mean C_t_ value (p=0.154) (Table 1). Storage of desiccated mosquitoes for up to six months had no effect on the ability to amplify parasite DNA. This result was confirmed by analysing the effect of time individually using a one-way ANOVA.

**Table 1:** **The effects of temperature and time on real-time PCR detection of*Plasmodium*DNA**

Study Condition	Partial Sum of Squares	Degrees of Freedom	F Statistic	P -Value
Model	169.45	12	1.77	0.062
Storage Time	42.84	3	1.78	0.154
Storage Temperature	97.49	3	4.06	0.009
Time*Temperature Interaction	18.83	6	0.39	0.883
Residual	912.02	114		
Total	1081.48	126		

Two-way factorial ANOVA analysis revealed no significant associations between time, or the interaction of time and temperature, on the ability to PCR detect *Plasmodium* parasites in infected mosquitoes.

Adjusted R-squared=0.068.

Number of Observations=127.

The average C_t_ values and confidence intervals for the temperature variable samples were 30.72 (29.42-32.03) for the -80°C temperature group, 28.69 (27.49-29.91) for samples stored at -20°C, 28.15 (27.19-29.1) for the 28°C group and 29.92 (28.11-31.34) for the 37°C group. The two-way ANOVA analysis (Table 1) revealed a significant effect of temperature on C_t_ (p=0.009). A subsequent one-way ANOVA was conducted to confirm the significance of the individual effect of temperature (p=0.012). This analysis demonstrated that only the -80°C and 28°C groups were significantly different from each other (p=0.013). The average C_t_ value of the 28°C group was 2.57 cycles less than the -80°C group, corresponding to approximately six times as much amplifiable DNA in these samples compared to those stored at -80°C. The 28°C holding condition improved the ability to detect parasite DNA by PCR, though a more general positive association between temperature and the ability to amplify DNA was not observed. The effect of temperature explained about 8% of the total variance (adjusted *R*^2^=0.079) observed in the individual statistical model.

The interactive effect of time and temperature on C_t_ value was not significant (p=0.883) (Table 1), indicating there is no interactive effect of these conditions on the amount of parasite DNA detected in desiccated mosquitoes. This result was confirmed using a one-way ANOVA.

## **Discussion**

*Plasmodium* DNA in desiccated mosquitoes was consistently amplified from all storage treatment groups despite DNA losses, in detection or yields, observed in other studies. No differences in parasite DNA detection by real-time PCR were noted between treatment groups, with the exception of one temperature group, in which the ability to amplify parasite DNA improved. Therefore, for at least six months, cold chain maintenance of specimens (the gold standard) is not necessary for preserving field-collections for subsequent small base pair target PCR-detection of *Plasmodium* DNA in desiccated mosquitoes. As no negative relationship was demonstrated between the experimental conditions and the amount of detectable parasite target DNA, it is likely that *Plasmodium* parasites will remain detectable by PCR for much longer periods, even after exposure to relatively high ambient temperatures.

Despite the logical premise that low-temperature storage of desiccated mosquitoes would best facilitate the amplification of parasite DNA (yielding the lowest C_t_ values), the 28°C storage group retained nearly six times as much amplifiable template compared to samples stored at -80°C. Under sub-freezing conditions, biological decomposition processes and ultimately the DNA-cleaving enzymatic activity of nucleases were expected to be inhibited. It was, therefore, surprising to see such a positive effect on DNA amplification from specimens stored at a warmer temperature. It has previously been demonstrated that high temperatures increase the susceptibility of DNA to shearing [9]; subsequent DNA isolation by precipitation methods may result in diminished yields as small DNA fragments are more readily lost in this process. It is possible that the methodology adopted here circumvents losses in PCR detection due to two factors: 1) a DNA carrier was used which facilitates extraction yields during precipitation; and 2) a small base pair template sequence was targeted (111 base pairs) for amplification. Even significant amounts of shearing therefore, may not have affected a small template target (commonly used for real-time PCR studies) and loss of small fragments would have been minimized. Furthermore, though there is no evidence to suggest that the PCR-targeted region is more shear or decay-resistant than other similar-length regions within *Plasmodium spp*. genomes, one limitation of this study is that this factor cannot be ruled out.

As the effect of temperature was not consistent across experimental groups, differences may reflect some variability in sample desiccation. It was noted that mosquitoes stored at temperatures above -80°C appeared drier and more friable at the time of dissection. If prior to storage, the desiccation process was not entirely complete, mosquitoes held at warmer temperatures may continue to desiccate, facilitating DNA preservation and perhaps extraction of DNA from tissues. Samples stored at -80°C may have experienced greater exposure to active nucleases while cooling, but before enzymes became temperature inactivated, and then again upon thawing for laboratory processing. Ice-crystal formation during the freeze-thaw cycle may also have damaged the integrity of DNA, although no definitive evidence exists to confirm this in this study. Though the -20°C and 37°C experimental groups were not statistically different, all groups stored above -80°C experienced on average less DNA degradation. This suggests that desiccation is critical to DNA preservation, and is perhaps more important than storage temperature.

The statistical analyses suggest no consistent association exists between the variables studied and the ability to detect parasites. Furthermore, little of the variation observed statistically was explained by the effects of time or temperature. Other factors that may influence parasite detection may be related to minor handling differences associated with DNA extractions, and dissections conducted at different time points. Though perhaps less significant in laboratory work, storage differences are likely magnified in field settings and in temporary lab facilities where sterile processing techniques and ease of sample manipulation are not easily attained. Therefore, though parasite DNA persisted in the lab irrespective of time and temperature treatment, the importance of proper handing techniques should not be underestimated in the field, and will likely require additional (and more rigorous) investigations.

In addition to its an application in malaria research, PCR-based assays are commonly used to detect other vector-borne parasites in arthropod hosts. Different insects, however, require different preservation techniques [15-17,23-26]. Mosquitoes and most dipterans, for example are quite small and desiccate rapidly, but for larger insects this medium is less effective with regard to successful PCR amplification [9]. Presumably, DNA-destructive nucleases remain active for longer periods when desiccation occurs slowly.

This research and other studies [9] suggest that desiccation of small dipterans, like mosquitoes, is an effective means of preserving field collections and the DNA of their endoparasites. Combined with efficient DNA extraction methods and small-target PCR detection, the results suggest that the methods described herein are appropriate for the detection of malaria parasites in field-collected, desiccated vectors. Furthermore, desiccation storage is highly amenable to field work. Drierite®, the desiccant used in this study costs just under $19 USD for 5 pounds, enough for over 6,000 sample preparations, a little less than a third of a penny per sample tube [27]. Furthermore, it can be “refreshed” in conventional ovens and reused. It is not toxic to skin, and not combustible, making it easy to travel with, and it can be used correctly by anyone without need for detailed training. Taking costs, logistics and effectiveness into consideration, desiccants combined with detection methods such as those described above should be standard for operational vector work in malaria studies.

## **Conclusions**

These data suggest that storage time and temperature do not diminish the ability to detect *Plasmodium* DNA in desiccated mosquito vectors by short sequence targeted PCR, for at least six months. Therefore, rigid maintenance of cold chain storage is unnecessary when storing mosquito specimens over desiccants. As desiccants offer an effective, inexpensive and logistically simple alternative to many preservation techniques currently in use, it can be confidently adopted for standard use in field research studies.

## **Competing interests**

The authors declare that they have no competing interests.

## **Authors' contributions**

MAR, BDB, and KAC contributed to the design of the study. MAR conducted the experiments with assistance from BDB and KAC. MAR and JK conducted the statistical analyses. MAR drafted and edited the manuscript based on critical revisions of all the authors. DMW and KAC provided overall direction and support for the study. All authors read and approved the final manuscript.
